# Advanced electrode design enables homogeneous electric field distribution for metal deposition studies via *in situ* liquid cell TEM

**DOI:** 10.1016/j.isci.2024.111119

**Published:** 2024-10-09

**Authors:** Xin Wei, Michael Noyong, Ulrich Simon

**Affiliations:** 1 Institute of Inorganic Chemistry (IAC), RWTH Aachen University, Landoltweg 1a, 52074 Aachen, Germany

**Keywords:** electrochemistry experimental methods, materials science, simulation in materials science, materials characterization, materials design

## Abstract

*In situ* liquid-phase electrochemical transmission electron microscopy (ec-TEM) as a valuable technique has been widely used in studying metal deposition in battery materials. While real-time observations of metallic nucleation, growth, and dendrite formation using microscale ec-TEM liquid cells are investigated, existing cells exhibit nonuniform electric field distribution along electrodes, limiting measurement reliability and quantitative analysis. Here, we introduce an advanced electrode design for ec-TEM chips, ensuring a uniform electric field for precise characterization of early-stage metal deposition closer to practical battery conditions. Both simulation and experimental investigations demonstrate that these specially designed ec-TEM chips facilitate quantitative electrochemical characterization combined with the *in situ* TEM technique in comparison with commercially available chips. We thus provide a significant progression toward optimizing the performance and reliability of quantitative *in situ* liquid-phase TEM measurements, essential for understanding and improving electrochemical systems.

## Highlights

• Advanced electrode design for ec-TEM chips ensures a homogeneous electric field
• Simulation confirms improved electric field distribution over commercial chips
• *In situ* liquid phase ec-TEM validated the improvement in electric field uniformity
• Optimization enhances reliability in studying nanoscale electrochemical processes

## Graphical abstract

**Figure:**
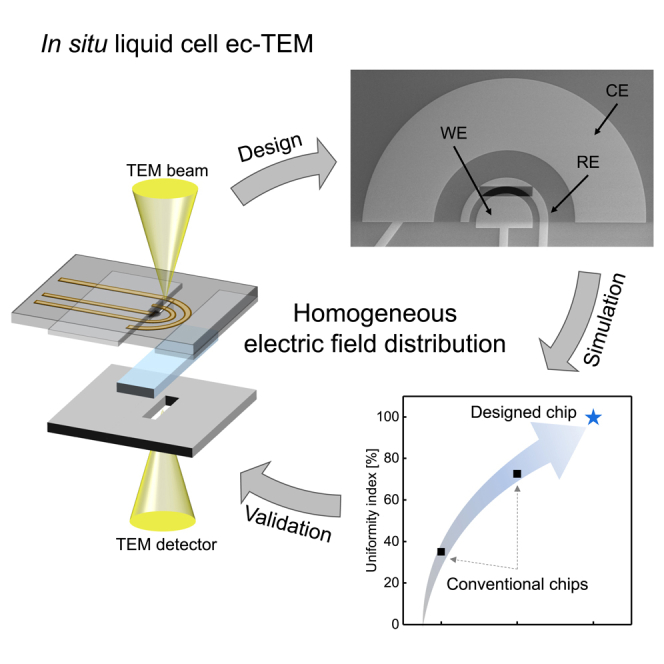

## Introduction

The formation of metallic dendrites on the anode during the charging-discharging process in electrochemical storage devices poses significant safety challenges due to short-circuits and thermal runaway incidents.^1^^,^^2^ Therefore, understanding the formation of early-stage metallic dendrites and of the solid electrolyte interphase (SEI) at the electrode/electrolyte interface is paramount for enhancing the performance and safety of batteries.^3^ Many efforts have been devoted to investigating and observing metallic dendrite formation, primarily by electrochemical tests^4^ and *ex situ/in situ* spectroscopy or microscopy techniques,^5^^,^^6^ however, with very limited structural information of micro-nanoscale dendrites due to the limited spatial resolution.^7^

*In situ* electrochemical transmission electron microscopy (ec-TEM) equipped with liquid electrolytes offers valuable nanoscale insights into the metallic deposition process during the electrochemical cycling, closely mimicking the real application scenarios with high spatial resolution.^8^^,^^9^^,^^10^^,^^11^^,^^12^^,^^13^ This technology has proven effective in observing the electro-deposition and -degradation in various systems, including catalyst deposition and metal batteries, across both aqueous and non-aqueous electrolytes. Advances in microscale electrochemical systems for *in situ* liquid-phase TEM has enabled detailed investigations of a diverse range of aqueous systems, such as copper,^14^^,^^15^ lead,^16^^,^^17^ nickel,^18^ metal-alloy,^19^^,^^20^^,^^21^^,^^22^ gold,^23^^,^^24^^,^^25^ magnesium,^26^ germanium,^27^ and zinc.^28^^,^^29^^,^^30^ Additionally, recent developments in *in situ* liquid-phase ec-TEM have enhanced the understanding of electrochemical processes by revealing chemical, morphological, and structural evolutions of the catalysis during the electrocatalysis at the nanoscale.^31^ For non-aqueous electrolytes, the deposition and dissolution of lithium dendrites were successfully observed during charging-discharging cycles to study the real-time dynamics of dendritic growth.^32^^,^^33^^,^^34^ Leenheer et al.^35^ documented the evolution of lithium deposit structure, transitioning from large grains to numerous small needle-like crystals while increasing the current densities from 1 mA/cm^2^ to 25 mA/cm^2^ within a custom microfabricated liquid cell. Holtz et al.^36^ explored the distinctive capability to monitor the real-time lithiation and delithiation dynamics through valence energy loss spectroscopy and the *ab initio* nonlinear response theory. In their work, a commercially available liquid cell featuring a bar-shape with a round edge working electrode surrounded by the arc-shaped reference and counter electrodes was utilized in the *in situ* ec-TEM tests. By employing similar liquid cells, Sacci et al.^37^ observed unique dendritic morphology of primary lithium deposits on the glassy carbon, while the secondary deposits exhibited a more varied range of morphologies. Nevertheless, this configuration has been shown to result in a nonuniformity of current density distribution and metal deposition through both simulation and experimental investigations.^19^

Furthermore, Mehdi et al.^38^ introduced an innovative approach for identifying the lithium presence from Li-containing compounds based on the density difference among the materials. This method offers a means for quantitative analysis during both lithium electrodeposition and electrolyte decomposition processes. However, the use of the liquid cell exhibits an obstacle to the potential quantitative analysis. The cell featuring a comb-shaped electrode configuration reveals nonuniform electric field distribution along the working electrode, as evidenced through the simulation and experimental observations, leading to a notable concentration of lithium growth at the electric field’s hot spot. Moreover, Sacci et al.^39^ observed a similar phenomenon of lithium dendritic nonuniformity and experimentally confirmed the dendritic growth influenced by the electric field and current distribution gradients.

Hence, the prevalent configurations of the liquid cell for *in situ* ec-TEM measurement unfortunately do not yield a homogeneous distribution of the electric field. This leads to an uneven formation of dendrites, which is unfavorable for the quantitative monitoring and analysis of dendrite growth. Despite numerous efforts to enhance the testing accuracy of the *in situ* liquid-phase ec-TEM measurement,^40^^,^^41^^,^^42^^,^^43^ prior reports have not yet focused on the development of electrode configurations to achieve quantitative analysis based on the observation of dendrite formation and accurate acquisition of current density.

In our study, we present an innovative electrode design aimed at promoting a uniform electric field distribution, enabling more precise microscopic quantitative characterization of early-stage lithium deposition, thus advancing its applicability to real-world battery scenarios. Through COMSOL 3D simulations, we quantitatively compared the performance of the proposed cell with conventional designs, illustrating a marked enhancement in the homogeneous distribution of current density along the electrode/electrolyte interface. Additionally, experimental investigation employing silver as the indicator and the designed ec-TEM cells confirmed the homogenous dendrite distribution and consistency of their growth behavior.

## Results

### Design of electrode configuration

**Figure 1:**
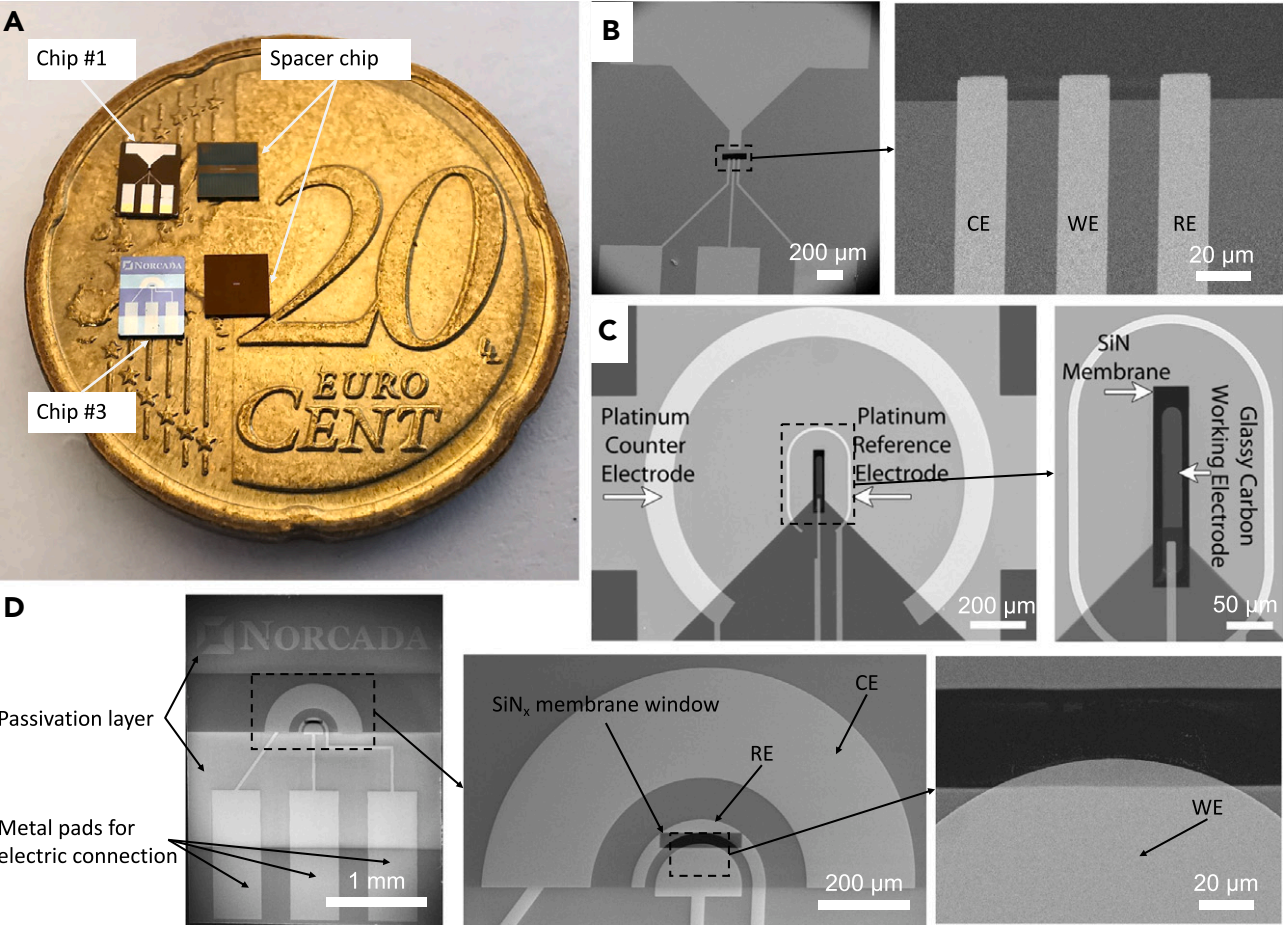
Demonstration of three types of electrode configurations (A) Photograph of commercially available electrochemical liquid cell Chip #1 (upper left), its spacer chip (upper right), and designed Chip #3 (bottom left), and its spacer chip (bottom right). (B) SEM image of Chip #1 and zoom-in on the three comb-shaped platinum electrode and the SiN_x_ membrane as the observation window. (C) SEM image of commercially available electrochemical liquid cell Chip #2 and zoom-in on the working and reference electrodes area of the chip. Reprinted with permission from Sacci et al.^37^ Copyright 2015, American Chemical Society. (D) SEM image of Chip #3 and zoom-in on the semicircular electrodes and the edge of working electrode in the middle of SiN_x_ membrane observation window.

Liquid cell for *in situ* TEM tests is constructed using two silicon chip substrates featuring silicon nitride (SiN_x_) membranes, each measuring 50 nm in thickness and functioning as observation windows. One of the silicon substrates is adorned with electrode patterns, designated as the electrode chip (Figure 1A left), while the other serves as the spacer chip (Figure 1A right). The electrode configuration in this study specifically emphasizes the electrode patterns situated on the electrode chips. The electrode configuration proposed in this work is based on the modification and improvement of two commercially available chips, referred to as Chip #1 ^10^^,^^39^ and Chip #2,^19^^,^^20^^,^^36^ which are widely employed in *in situ* liquid-phase ec-TEM studies. Figures 1A and 1B demonstrates the photo and the schematic illustration of Chip #1 developed by Hummingbird Scientific (Lacey, WA, US), showcasing a comb-shaped electrode layout. This configuration consists of three parallel platinum bar electrode patterns, with their terminal ends emerging on the SiN_x_ membrane, which serves as the observation aperture. In contrast, Chip #2 (Figure 1B) from Protochips (Morrisville, NC, US) features a distinctive electrode design, consisting of a bar-shaped electrode with a rounded tip serving as the working electrode. Positioned centrally, this working electrode is encircled by arc-shaped electrodes functioning as reference and counter electrodes, outlining a different pattern compared to Chip #1.

The electrode pattern of the designed chip (the bottom left chip in Figure 1A), denoted as Chip #3 hereafter, adopts the architectural framework of Chip #1 to ensure compatibility with established commercial devices in real-world applications. In this configuration, a centrally located half-round platinum pattern is encircled by two semicircular rings of varying widths and radii, as shown in Figure 1D. These 50 nm-thick concentric semicircles function respectively as the working electrode (WE), counter electrode (CE), and reference electrode (RE) elements. This configuration allows the improvement of the homogeneity of the electric field distribution within the microscale liquid cell.^44^^,^^45^ Moreover, the observation window deliberately maintains a narrower width of 30 nm to minimize the bulging effect during real applications in TEM high-vacuum conditions.^46^^,^^47^^,^^48^ Most notably, the majority of the half-round WE is positioned beneath the observation window, with only its upper edge visible through the aperture. This strategic placement is intentional, aiming to mitigate excessive pressure exerted on the surface of SiN_x_ membrane, thereby minimizing the risk of rupture. In addition, 100 nm-thick passivation layers have been applied with dual functions: to prevent additional conductive connection wires from contributing unwanted currents and to act as spacers within the system. These custom-designed chips are manufactured by Norcada (Edmonton, AB, Canada).

### COMSOL 3D simulation

**Figure 2:**
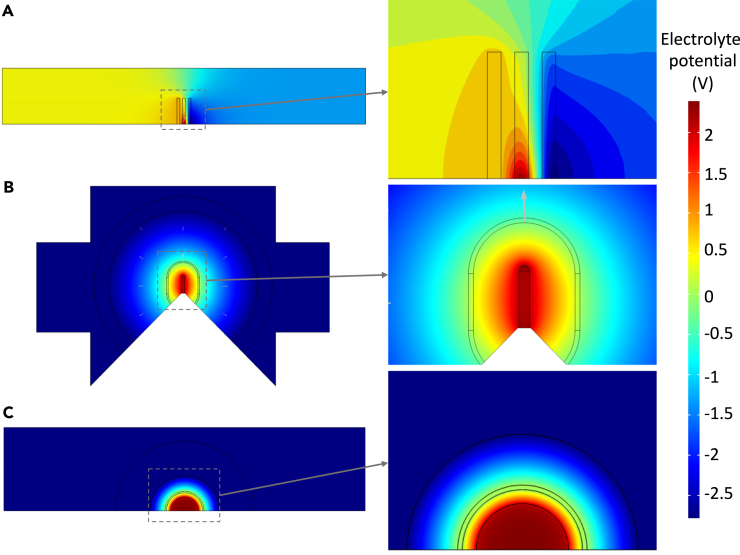
Analysis of COMSOL simulation results for three configurations The color counter-mapping and zoom-in images showcasing the electrolyte potential distribution in the models of three electrode configurations: (A) Chip #1, (B) Chip #2, (C) Chip #3. The legend provides the corresponding electrolyte potentials represented by different colors.

According to the given initial simulation condition, the COMSOL Multiphysics 5.6 simulation was conducted to model and visualize the effect of the electrode configurations on the electric field distribution for three-type chips. Figure 2 displays the contour plots of the electrolyte potential distribution as obtained by the simulations on the electrode configurations. The color index of the contour represents the local electrolyte potential value, where red indicates a high electrolyte potential of 2.5 V. To enhance clarity, only the upper side of the cell chamber is presented, given the negligible differences resulting from the ultrathin thickness. Figure 2A demonstrates the electric field distribution in Chip #1 equipped with three comb-shaped electrodes, the red region around the working electrode corner facing the counter electrode, indicating the electric field concentrated at the rectangle corner and end.

The counterplot of the commercially available Chip #2 (Figure 2B) presents the red area as having high electrolyte potential at the tip end of the bar-shaped working electrode, indicating a higher electrolyte potential in that region, which is exactly located at the observation window. The simulated electrolyte potential maps indicate that the top-tip area is more likely to generate higher local potential and increase the dendrite formation rate. This suggests that dendrite formation occurs earlier than in the rest region where the same potential contribution was expected. It misleads the accurate determination of the applied potential for dendrite growth. In contrast, the electrode configuration of the designed Chip #3 (Figure 2C) demonstrates a homogeneous red region distributed along the half-round-shaped working electrode, a significant improvement in the potential distribution, owing to its concentric-semicircle structure. The simulation outcomes underscore a notable enhancement in the uniformity of potential distribution achieved with the designed electrode structure.

**Figure 3:**
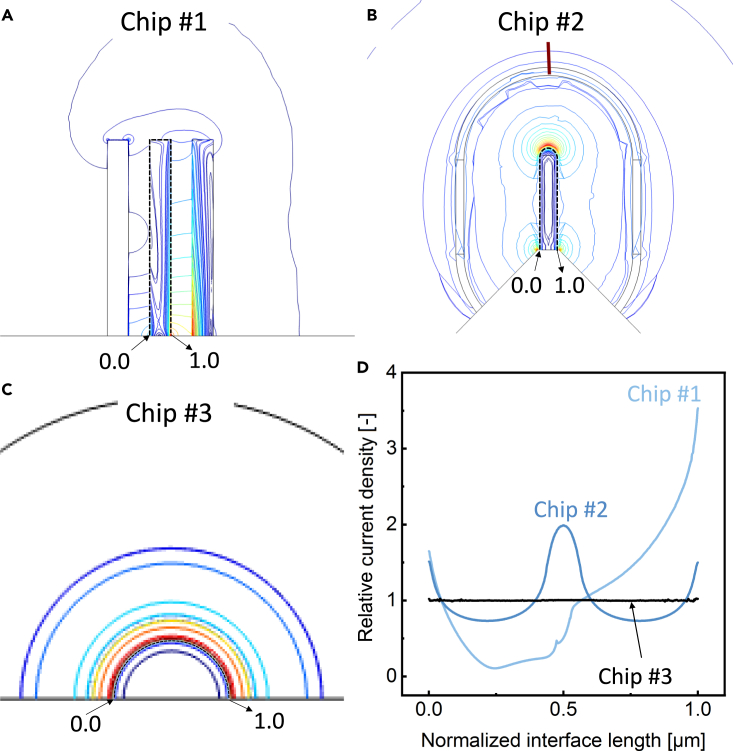
Quantitative analysis of current density distributions for three configurations The electrolyte current density magnitude plots of (A) Chip #1, (B) Chip #2, and (C) Chip #3. (D) Relative current density distributions along the electrode/electrolyte interface as a function of normalized interface length in the models.

Moreover, previous studies have shown that dendrite formation exhibits a pronounced dependence on local current conditions originating from surface irregularities caused by factors, such as the morphology of the working electrode, defects in the SEI, and relative asymmetry in deposition and stripping rates.^49^ The growth rate of dendrites demonstrates a unidirectional increase with escalating current densities.^50^^,^^51^^,^^52^ Additionally, it has been observed that under an equivalent total deposited charge, lower local current densities facilitate the formation of planar metal growth.^53^ Conversely, higher local current densities promote the growth and development of dendrite arms, driven by the dominance of electrodeposition rates.^53^ Therefore, an investigation into the local current at the interface between the working electrode and electrolyte was conducted to assess the impact of electrode configuration on dendrite growth. Figures 3A–3C displays the distinctive magnitude plots of various electrolyte current densities obtained from three configurations, which indicates Chip #3 stands out for achieving the most homogeneous distribution of current density.

Furthermore, the current density distribution, extracted at the electrode/electrolyte interface from three distinct models, is visualized in Figure 3D. The resulting values of current densities, normalized by their respective averages, are represented as a function of normalized interface length. The current density distribution obtained from these configurations differs significantly. Chip #1 exhibits a highly uneven current density distribution with a uniformity index of 35.0%. The relative current density on the side facing RE is significantly lower than 1 (the average), whereas the current density near the boundary with CE gradually increases. Chip #2 displays a symmetric current density distribution on both sides, with higher current density observed at the edges and the central tip region, achieving a uniformity index of 72.6%. In contrast, Chip #3 demonstrates a noticeable improvement in current density distribution with a significant homogeneity with a calculating uniformity index of 99.7%. It implies dendrite growth along the electrode occurs under consistent conditions. These results underscore the advancement achieved in the electrode configuration of the designed chip.

### *In situ* liquid-phase electrochemical TEM test

To validate the simulation results, *in situ* electrochemical TEM tests were performed in the liquid cell. Silver deposition was chosen as the indicator due to its superior observability and operational simplicity compared to lithium.^54^ This process was conducted using a 2 mM silver nitrate (AgNO_3_) and 50 mM sodium nitrate (NaNO_3_) aqueous electrolyte. The scanning voltage range spanned from ˗500 to 50 mV, with a scanning rate of 10 mV/s. Chips #1 and #3 were specifically selected for these tests based on their compatibility with the device. Although Chip #2 could not be experimentally evaluated in this study due to incompatibility with our experimental setup, insights from prior research by Mehdi et al.^38^ and Sasaki et al.^55^ offer valuable context. These studies, which utilized the same or similar configuration of Chip #2, have demonstrated that metal preferentially deposits at the round tip of the bar-shape electrodes. These observations align with the COMSOL simulation results presented in Figure 3D.

**Figure 4:**
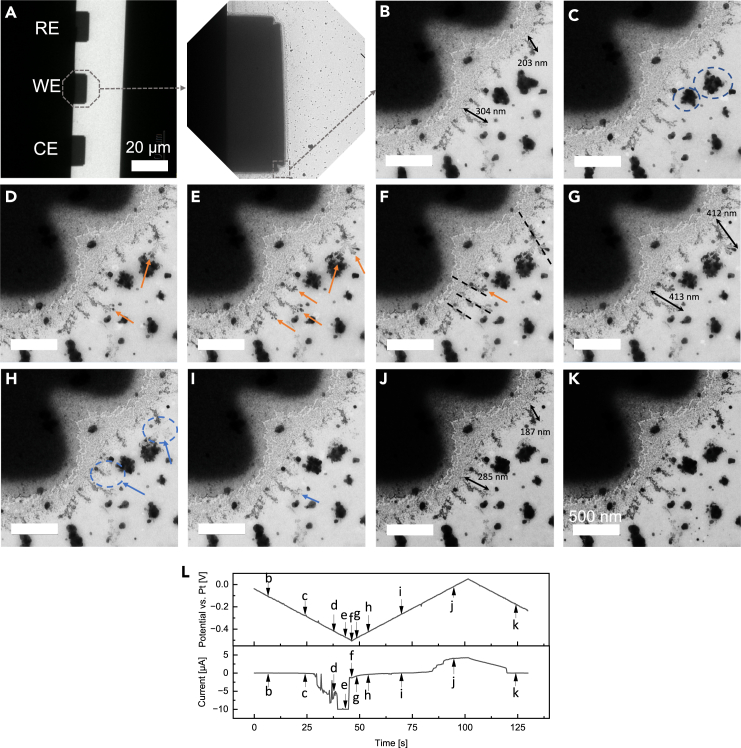
Silver deposition and dissolution behaviors on the rectangular electrode of Chip #1 (A) The TEM survey image of comb-like electrodes on Chip #1 within the observation window and the zoom-in image of the working electrode. Scale bar, 20 μm. (B–K) Time evolution of the growth and dissolution of Ag dendrites during the CV measurement following 5 CV cycles using Chip #1. The dendrite lengths were measured via DigitalMicrograph. Scale bar, 500 nm. See also Video S1. (L) The corresponding applied electric potential and measured current from frame a to frame j. The black arrows highlight the length values of the specific dendrites. The orange and blue arrows highlight the growth and dissolution of dendrites, respectively.

Figure 4 displays a series of representative frames depicting silver electrodeposition using Chip #1, extracted from the *in situ* ec-TEM video sequence during the CV measurement following 5 CV cycles (see also Video S1). Figure 4A displays a TEM survey image of three electrodes within the observation window, along with a zoom-in image from the intact tip of the working electrode. Notably, the zoom-in image shows that the rectangle Pt electrode has irregular concave corners forming two small convex parts, which can result in additional locally high current density. This observation has been found in previous studies based on microchips from the same manufacturer.^10^^,^^39^^,^^56^^,^^57^ Notably, dendritic structures, remnants from prior charging/discharging processes, are observed at the corner of the platinum working electrode (Figure 4B). Specifically highlighted are two dendrites with lengths marked at 203 nm and 304 nm. The dead silver metal particles (indicated by dashed circles in Figure 4C) undergo morphological changes under the influence of the electric field. As the voltage sweeps from ˗100 mV to ˗500 mV, the dendritic structure irregularly extends radially outward (Figures 4D–4G), as indicated by orange arrows marking their growth and directions. An intriguing observation emerges as the upper dendrite branches irregularly into two directions, influenced by forces from both the dead silver particle and the electric field. The previously marked dendrites exhibit notable growth, measured at lengths of 412 nm and 413 nm, respectively (Figure 4G). Certain dendrites preserve the shape and then localized silver stripping occurs at the tip of the dendrite structure (marked by dashed circles in Figures 4H–4J) upon sweeping the voltage back from ˗400 mV to 50 mV, featuring the decreasing lengths measured at 187 nm and 285 nm. Furthermore, artificial markings (dash lines in Figure 4F) emphasize the variable morphology of individual dendritic grains emanating from randomly oriented edges of the platinum electrode, likely due to the nanoscale inhomogeneity of the electric field and current distribution caused by the irregular electrode configuration.^58^ These observations align with previous literature reports on lithium deposition processes.^39^

Video S1. Silver deposition and dissolution behaviors on Chip #1, related to Figure 4

**Figure 5:**
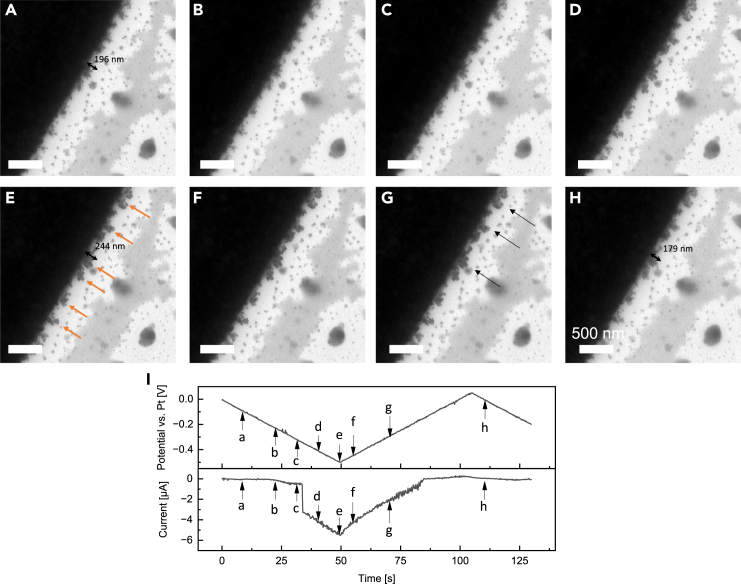
Silver deposition and dissolution behaviors on the semicircular electrode of Chip #3 (A–H) Time evolution of the growth and dissolution of Ag dendrites during the CV measurement following 5 CV cycles using Chip #3. The dendrite lengths were measured via DigitalMicrograph. Scale bar, 500 nm. See also Video S2. (I) The corresponding applied electric potential and measured current from frame a to frame h. The black arrows highlight the length values of the specific dendrites, and the orange arrows highlight the growth of dendrites.

In contrast, the silver electrodeposition using Chip #3 presents a distinct phenomenon, as illustrated in Figure 5, showcasing a series of representative frames obtained from the *in situ* ec-TEM video sequence following 5 CV cycles (see also Video S2). Noteworthy are the dendritic remnants from previous CV processes observed at the electrode/electrolyte interface (Figure 5A), featuring a mossy structure with a maximum length of 196 nm. Subsequently, the growth of dendrites on Chip #3 extends to form broccoli-like structures by sweeping the voltage from 0 mV to ˗500 mV, as indicated by the orange arrows (Figures 5B–5F). The maximum dendritic length is measured at 244 nm, which then decreases to 181 nm (Figure 5H) as the applied voltage is swept back to ˗50 mV. Unlike the observations with Chip #1, the silver deposits on Chip #3 have a mossier morphology with short and reversible dendrite formation, demonstrating significant and consistent dendrite growth behavior and morphology. These findings with Chip #3 suggest the achievement of a homogeneous electric field along the electrode, contributing to the observed differences in morphology. Nevertheless, it is worth underlining that the local environments during the individual *in situ* ec-TEM tests can vary considerably depending on factors such as the local pH, the presence of oxygen, and the stability of electrolytes under electron irradiation.^30^^,^^32^^,^^59^^,^^60^ Consequently, to achieve reliable quantitative comparisons of dendrite growth sizes across different setups, more precise control over local operational conditions and the selection of stable indicator systems are essential.

Video S2. Silver deposition and dissolution behaviors on Chip #3, related to Figure 5

**Figure 6:**
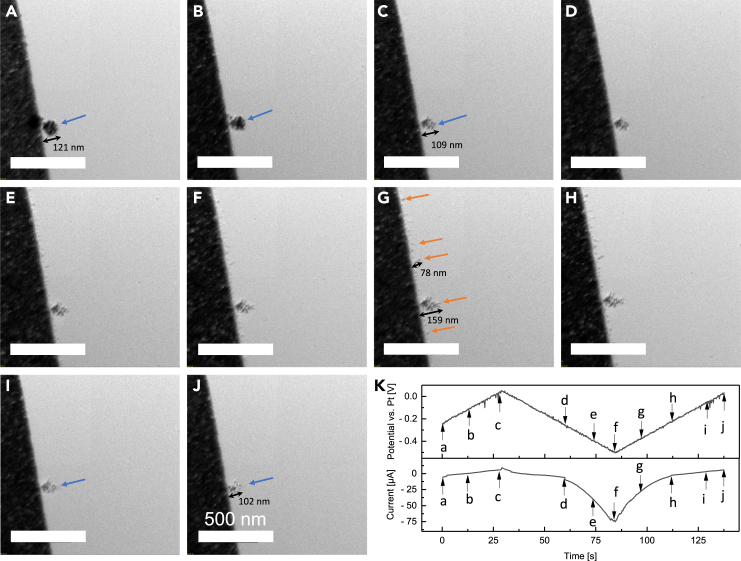
Early-stage deposition and dissolution behaviors of silver on the semicircular electrode of Chip #3 (A–J) Time evolution of the formation and dissolution of Ag dendrites during the CV measurement using Chip #3. The dendrite lengths were measured via DigitalMicrograph. Scale bar, 500 nm. See also Video S3. (K) The corresponding applied electric potential and measured current from (A) to (J). The black arrows highlight the length values of the specific dendrites. The orange and blue arrows highlight the growth and dissolution of dendrites, respectively.

Moreover, the initial formation of silver dendrites was further investigated on Chip #3 during the initial cycle of CV measurement. Figure 6 illustrates sequential *in situ* TEM images representing the formation, subsequent growth, and dissolution of silver dendrites acquired using Chip #3 for the liquid cell TEM test (see Video S3). Figures 6A–6C displays the Pt electrode appears smooth without obvious dendrite while the voltage sweeps from ˗250 mV to 0 mV at a scan rate of 10 mV/s. Meanwhile, an accidentally involved particle undergoes dissolution, decreasing in size from 121 nm to 109 nm (highlighted by the blue arrow). Subsequently, the fine needle-like structures begin to emerge on the electrode surface as the CV scanning progresses in the negative direction down to ˗500 mV (Figures 6D–6F), indicating the initial nucleation of dendritic structures. Instantaneously, the mossy dendrites grow rapidly and aggregate to form larger branches (with a maximum length of 78 nm) which were evenly distributed along the electrode, as the voltage is swept back to ˗500 mV. Remarkably, significant dendritic growth with distinct branches is observed on the marked particle, achieving a maximum length of 159 nm (Figure 6G). Finally, the dissolution of silver dendrites occurs as the voltage is swept back to 50 mV (Figures 6H–6J), resulting in a smooth Pt surface demonstrating the complete reversible dissolution of Ag dendrites. Additionally, the particle size is reduced from 159 nm to 102 nm.

Video S3. Silver deposition and dissolution behaviors on Chip #3, related to Figure 6

**Figure 7:**
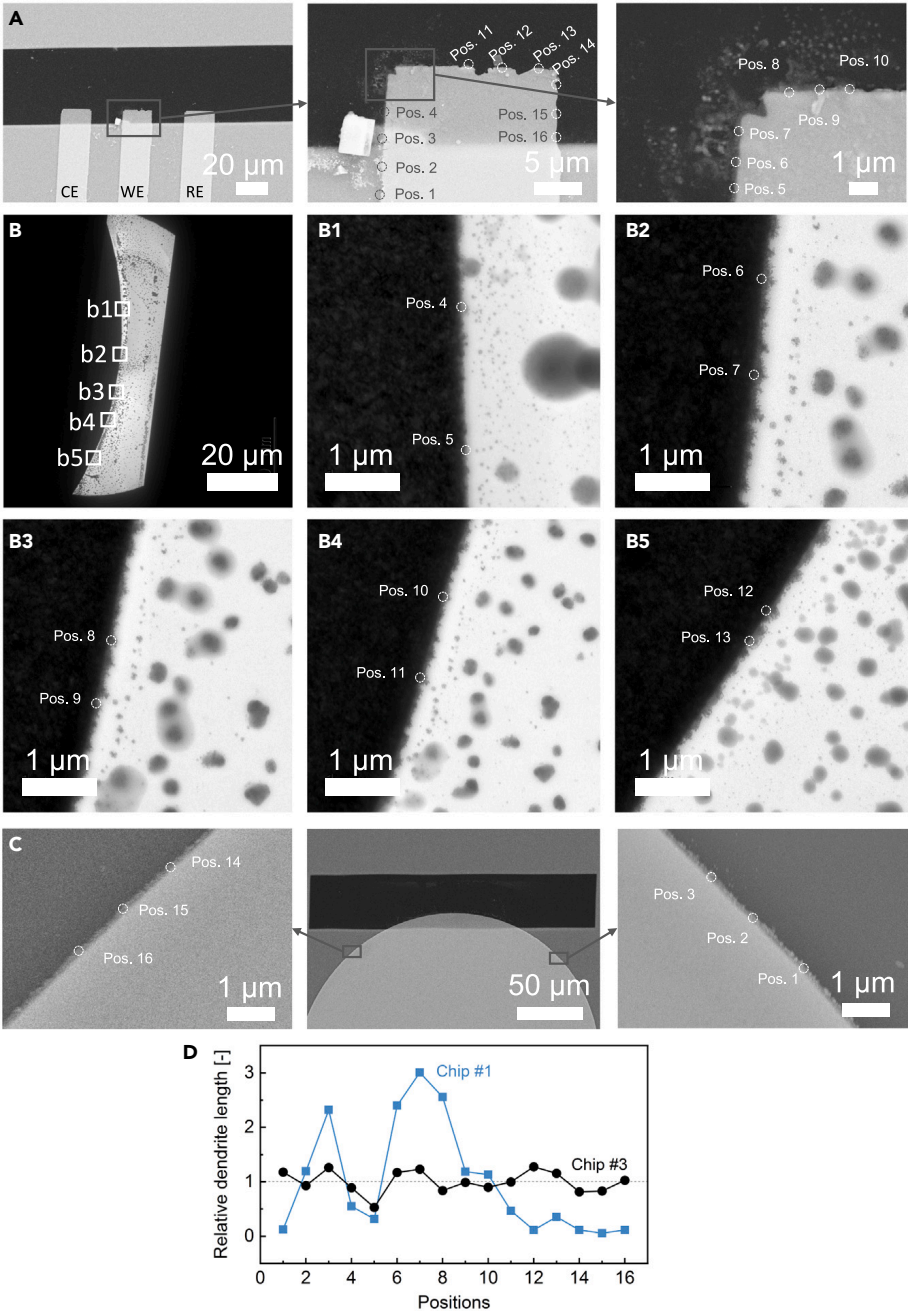
Postmortem analysis of Chip #1 and #3 after *in situ* electrochemical TEM tests (A) Postmortem SEM images acquired from Chip #1 accompanied by the respective zoom-in images. (B) TEM survey images of Chip #3 directly after the CV measurements and zoom-in images at different spots on Chip #3 (B1-B5). (C) Postmortem SEM images acquired from Chip #3 accompanied by the respective zoom-in images. (D) Relative dendrite lengths distributed along the electrode/electrolyte interface, measured via DigitalMicrograph at 16 different positions for both Chip #1 and Chip #3 (as indicated by the white dashed circles in images A–C).

The postmortem analysis of the liquid cells following *in situ* TEM tests revealed direct insights into dendrite distribution along the WE. In Figure 7A, scanning electron microscope (SEM) images illustrate silver deposition surrounding the rectangular WE on Chip #1. Post cycling, the WE corner near the CE exhibited predominantly silver depositions, while the side adjacent to the RE displayed a clean field and edge. This disparity suggests an uneven preference for dendrite formation and growth along the rectangle configuration of WE.

Moreover, a TEM survey of Chip #3 directly after *in situ* TEM tests involved randomly selecting 5 spots, as shown in Figure 7B. The silver deposition showcased a consistent mossy microstructure with similar lengths across these spots. A similar phenomenon is characterized by postmortem analysis along the WE except for the area on the observation window (Figure 7C). Additionally, dendrite lengths were measured from the postmortem images for a quantitative analysis. Sixteen different positions along the electrode/electrolyte interface of Chip #1 and Chip #3 were selected, as indicated by the white dashed circles in Figures 7A–7C. The relative dendrite lengths were calculated by dividing each individual length by the average value, providing a measure of the uniformity of dendrite growth distribution. Figure 7D presents the relative dendrite lengths for Chip #3 remains clustered around 1, implying minimal variation, whereas those of Chip #1 exhibit significant variability. The results indicate the representative behavior and homogeneous dendrite distribution along the WE regardless of the side effect of the electron beam. This comprehensive analysis provides valuable insights into the consistency and behavior of dendrites within the electrode structure post-testing.

## Discussion

The *in situ* electrochemical TEM tests provided validation for the simulation results, affirming the viability of the electrodes as designed. The achievement of uniformly distributed dendrites underscores the practical efficacy of the proposed design in enhancing electric field distribution. This advancement opens opportunities for wider applications of microscale *in situ* liquid-phase TEM cells. Furthermore, the scalability of this electrode design to other application systems shows considerable promise. The design principles—such as uniform electric field distribution and precise control over experimental parameters—can be adapted for various material systems beyond those tested. For instance, the design could be employed in studying advanced materials used in catalysis, environmental sensing, or even energy storage technologies across both aqueous and non-aqueous electrolyte conditions. The refined electrode architecture not only addresses issues related to potential fluctuations but also enables more precise control over experimental parameters, setting a foundation for improved experimental conditions in a wide range of application systems.

In conclusion, this study presents a significant advancement in microscale electrode configurations of liquid cell chips tailored for *in situ* electrochemical TEM measurements. Through rigorous COMSOL simulations, the designed electrode configuration has been demonstrated to markedly enhance the uniformity of electric field and current distribution. The experimental validation conducted through *in situ* liquid-phase TEM tests and the postmortem analysis, comparing commercially available chips with the designed one, has confirmed the latter’s capability to achieve evenly distributed, reversible, mossy microstructural dendrites along the whole electrode. This study signifies a noticeable progression toward optimizing the performance and reliability of quantitative *in situ* liquid-phase TEM measurements, providing valuable insights to the scientific community and setting the stage for further advancements in comprehending nanoscale phenomena.

### Limitations of study

We want to point out that the comparative analysis of the different chip designs is only possible through an identification parade of chips fabricated by different manufacturers. Hence, our focus is solely on the comparison of these chip designs, and not on the assessment or ranging of manufacturers, electrode materials, production processes, etc. Additionally, a direct experimental comparison between Chip #2 and Chip #3 is not feasible due to the incompatibility of Chip #2 with our current equipment.

## Resource availability

### Lead contact

Further information and requests for resources and reagents should be directed to and will be fulfilled by the lead contact, Xin Wei (xin.wei@ac.rwth-aachen.de).

### Materials availability

This study did not generate new unique reagents.

### Data and code availability

• All data reported in this paper will be shared by the lead contact upon reasonable request.
• No new code was generated during the course of this study.
• Any additional information required to reanalyze the data reported in this paper is available from the lead contact upon reasonable request.

## Acknowledgments

This work was funded by the 10.13039/501100002347German Federal Ministry of Education and Research (10.13039/501100002347BMBF) as part of the research cluster “AQua” within the project InOPlaBat (grant number 03XP0352A).

## Author contributions

X.W. conceived, performed, analyzed the research, and wrote the paper. U.S. supervised the study. U.S. and M.N. and revised the paper. All the authors discussed the results and commented on the manuscript.

## Declaration of interests

The authors declare no competing interests.

## STAR★Methods

### Key resources table

**Table:** 

REAGENT or RESOURCE	SOURCE	IDENTIFIER
**Chemicals, peptides, and recombinant proteins**		

Silver nitrate	Merck	CAS: 7761-88-8
Sodium nitrate	Merck	CAS: 7631-99-4

**Software and algorithms**		

DigitalMicrograph	Gatan, Inc.	www.gatan.com
Origin 2023	Originlab	www.originlab.com
COMSOL Multiphysics 5.6	COMSOL	comsol.com
NOVA 1.11	Metrohm	www.metrohm.com

### Experimental model and study participant details

This study does not use experimental methods typical in the life sciences.

### Method details

#### COMSOL 3D simulation

COMSOL simulations play a pivotal role in this study by enabling the visualization of the electric field within the microscale setups. Furthermore, these simulations facilitate the extraction of current density distributions along the interface of the working electrodes and electrolyte. To quantitatively assess the performance of the designed electrode configuration, the electrochemical 3D simulations of the various electrode systems were conducted. The model for the electrical characterization of TEM electrode systems is based on the COMSOL Multiphysics 5.6 commercial software package in this study. The AC/DC module contains generic physics interfaces for modeling potential and current distribution in electrochemical cells. The secondary current distribution interface is utilized for studying the transport of charged ions in an electrolyte of uniform composition as well as current conduction in electrodes using Ohm’s law in combination with a charge balance.

As shown in Figure S1 (in supplemental information), thin electrolyte layers of 100 nm were selected in all three models to approach the application conditions of real *in situ* TEM measurement. All electrodes in the modeling are standardized to be 50 nm thick and composed of platinum for comparability. The bulging effect of the SiN_x_ membrane is neglected to simplify the modeling process.^46^ The electrodes of Chip #1 have been simplified to the right-angled rectangles since the size of their irregular concave corners is negligible compared to that of the whole electrode. Notably, the geometrical parameters for Chip #2 were extracted from previous publications.^37^ The entire geometries were meshed (Figure S1 right row) using tetrahedral elements with maximum and minimum element sizes set at 91 and 0.01, respectively. To optimize computational efficiency, the edges of working electrodes were defined ultrafine. Boundary conditions were established with the counter electrode and reference electrode defined as grounded, while the working electrode was supplied with a potential of 3 V. For the electrolyte, LiPF_6_ in EC:DEC (1:1) was chosen to replicate the conditions relevant to lithium-ion battery applications.

The uniformity index of relative current densities is calculated based on the variance and the maximum possible variance via Equation 1.^61^^,^^62^:

(Equation 1) $$Uniformity\;Index=\left(1-\frac{\sum_{i=0}^{n}\left|I_{i}-I_{avg}\right|}{nI_{avg}}\right)\times 100\%$$

where n is the data number of the local current density, and I_avg_ is the average value of the current density. The higher value of the uniformity index indicates a more uniform distribution of current density along the electrode, while a lower value suggests a more concentrated or uneven distribution.

#### *In situ* ec-TEM measurement by silver

To validate the feasibility of the designed configuration and its impact on the dendrite formation, *in situ* ec-TEM measurements were conducted utilizing both commercial and designed chips. It is important to clarify that this work focuses exclusively on the comparison of design and does not consider variations in electrode materials or production processes. The characteristics of current commercial chips with respect to electrode preparation and quality may vary among manufacturers. For this investigation, Chips #1 and #3 (depicted in Figure 1A) were exclusively selected due to their compatibility with the equipment setup. Figure S2 provides exploded schematic illustrations of the liquid cell assembly by using Chip #1 and Chip #3, with a thin liquid electrolyte layer densely filling in the confined space between two chips post-assembly. The SiN_x_ membrane windows effectively insulate the electrolyte from the vacuum environment within the TEM column. The liquid cell was assembled within the *in situ* TEM liquid holder from Hummingbird Scientific (Lacey, WA, US). The metal pads of the electrode chips were aligned with the holder wires, ensuring a proper circuit connection to the potentiostat Autolab/PGSTAT101 (Metrohm/Eco Chemie, Utrecht, the Netherlands).

The *in situ* liquid cell ec-TEM experiments were conducted using Libra 200FE microscope (Carl Zeiss Microscopy GmbH) operating at an acceleration voltage of 200 kV. To mitigate electron irradiation damage, the electron beam current densities were carefully controlled during observations. The electrochemical deposition and dissolution processes occurring on the WE were recorded using a Gatan Ultrascan 1000 CCD camera, with each frame exposed for 0.5 s. Silver was chosen as the indicator owing to its high contrast and previously established feasibility for conducting *in situ* ec-TEM tests under atmospheric conditions.^54^ A solution comprising 2 mM silver nitrate (AgNO_3_) and 50 mM sodium nitrate (NaNO_3_) in water was used to deposit silver dendrites with precision. During the *in situ* TEM measurement, cyclic voltammetry (CV) was employed within a potential window ranging from 50 mV to −500 mV versus a platinum quasi-RE with a scan rate of 10 mV/s.

### Quantification and statistical analysis

The TEM images and videos were collected and analyzed by DigitalMicrograph software as Gatan Microscopy Suite. Data tabulation, analysis, and plotting were performed in Origin 2023 software.

### Additional resources

No relevant additional resources have been used to carry out this work.
