# Supplementary material for: Advanced electrode design enables homogeneous electric field distribution for metal deposition studies via in situ liquid cell TEM
Source: iScience. 2024 Oct 9;27(11):111119. doi: 10.1016/j.isci.2024.111119 (PMC11530855; doi:10.1016/j.isci.2024.111119)
Supplement: Document S1. Figures S1 and S2 [file mmc1.pdf]

iScience, Volume *27*

Supplemental information

Advanced electrode design enables homogeneous electric field distribution for metal deposition studies via *in situ* liquid cell TEM

Xin Wei, Michael Noyong, and Ulrich Simon

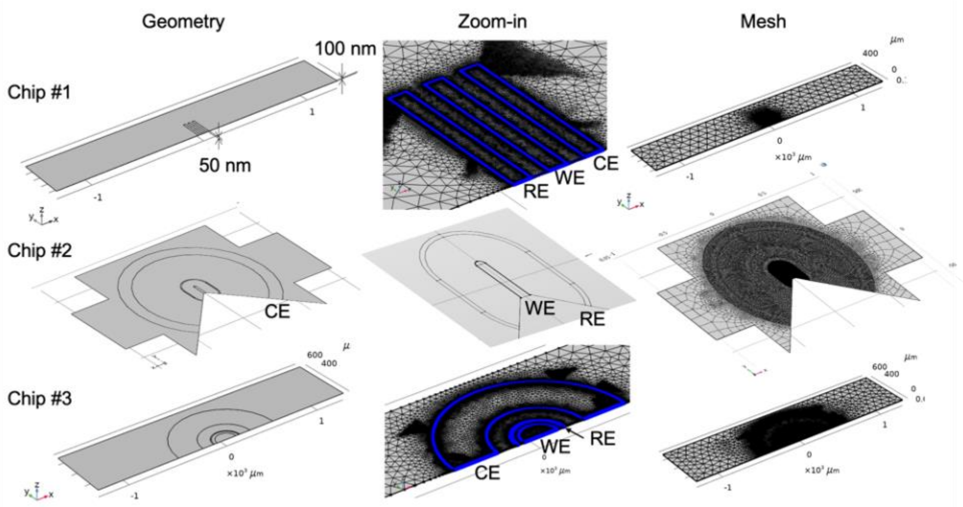

Figure S1. COMSOL simulation models for the three different electrode configurations The images of geometry (left row), zoom-in (middle row), and meshing (right row) for the COMSOL modeling on Chip #1 (upper line), Chip #2 (middle line), and Chip #3 (bottom line).

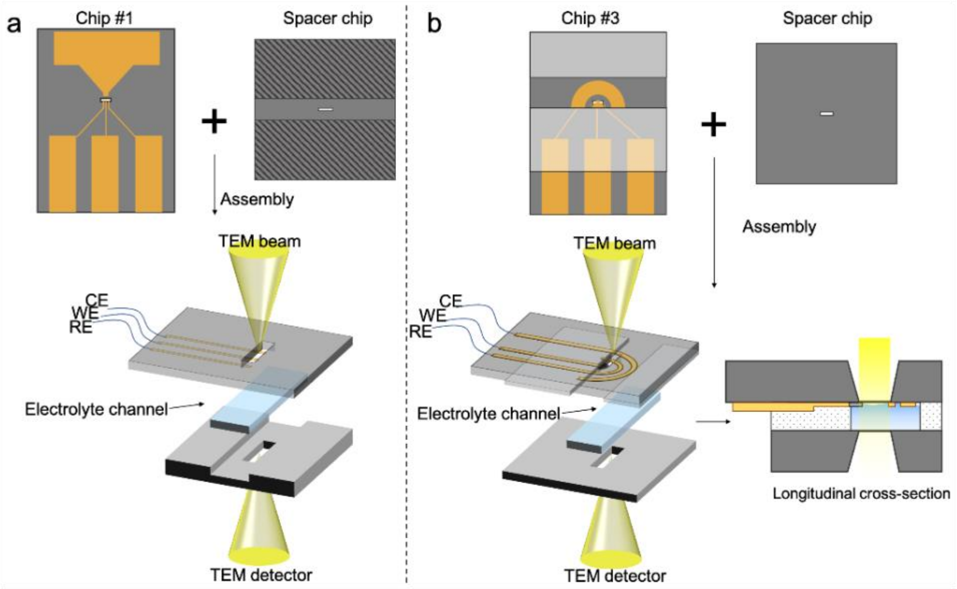

Figure S2. Illustration of liquid cell assembly

Exploded schematic of the liquid cell assembly and application for *in situ* ec-TEM tests by using (a) Chip #1 and (b) Chip #3.
